# Optimization of a Loop Mediated Isothermal Amplification (LAMP) Assay for In-Field Detection of *Dichelobacter nodosus* With *aprV2* (VDN LAMP) in Victorian Sheep Flocks

**DOI:** 10.3389/fvets.2019.00067

**Published:** 2019-03-08

**Authors:** Nickala Best, Grant Rawlin, Robert Suter, Brendan Rodoni, Travis Beddoe

**Affiliations:** ^1^Beddoe Laboratory, Department of Animal, Plant and Soil Science, La Trobe University, Melbourne, VIC, Australia; ^2^Centre for AgriBioscience (AgriBio), Department of Economic Development, Jobs, Transport and Resources, Victorian Government, Melbourne, VIC, Australia; ^3^Agriculture Services and Biosecurity Operations, Department of Economic Development, Jobs, Transport and Resources, Victorian Government, Attwood, VIC, Australia

**Keywords:** LAMP, in-field, footrot, ovine, *aprV2*, on-farm diagnostic

## Abstract

*Dichelobacter nodosus* is the primary etiological agent of footrot in sheep and has a variety of virulence factors. Of these, AprV2, an extracellular protease, has been shown to be capable of causing severe or “virulent” disease symptoms under the right conditions. Due to this, a loop-mediated isothermal amplification (LAMP) assay for the detection of *aprV2*-positive *D. nodosus* (VDN LAMP) was developed and evaluated for field use. A sample of 19 sheep flocks (309 sheep) in Victoria, Australia, were tested to determine the optimum conditions for in-field VDN LAMP assay use and sampling, for detecting *aprV2-*positive *D. nodosus* infected sheep. VDN LAMP performance was compared to a validated rtPCR that detects *aprV2* and the benign strain counterpart, *aprB2*, using biologically duplicate samples to determine sensitivity and specificity. Flocks were sampled either in winter-spring (moist) or early summer (dry) conditions and had a range of clinical expressions of the disease ovine footrot. Variables considered for optimizing field performance were: sample collection method, sample preparation, clinical expression of disease, and nature of the feet when sampled (moist vs. dry, clean vs. soiled). The test was found to perform best when sheep were sampled with moist, clean feet, using a dry swab with the sample prepared in alkaline polyethylene glycol, pH 13.0, as the collection buffer. A sensitivity of 89% and specificity of 97% was seen when used in-field under these conditions, when compared to *aprV2* detection by rtPCR, with “very good” agreement to rtPCR results. This study shows the VDN LAMP test is easy to use in-field to identify the presence of *aprV2-*positive *D. nodosus* in sheep flocks.

## Introduction

Footrot in sheep is a disease that causes considerable welfare and economic concerns. *Dichelobacter nodosus*, a gram-negative bacterium, is the primary etiological agent and causes the hard hoof to separate or “underrun” from the soft underlying tissue of the sheep foot (1). In Australia, clinical footrot is divided into two forms—benign and virulent. This is referring to the visual symptoms of *D. nodosus* infection and in Victoria is based on a modified Egerton scoring system of 1–5 (2). In Victoria, a flock is considered to have clinically virulent footrot when 1% or more of the inspected flock have score 4 or 5 lesions, while scores of 1 and 2 are indicative of benign footrot and show symptoms resembling interdigital dermatitis (3). Footrot is easily spread over the pasture, particularly in Spring in Victoria (September–November), posing a biosecurity threat both on the farm between flocks and in the larger industry (4, 5).

The clinical severity of the disease is an interplay between sheep genetics (resistance), bacterial genetics (strain virulence), and environmental conditions (6). Two extracellular protease genes, *aprV2*, and *aprB2* have been shown to correlate to the clinical severity of disease (7, 8). Of these, a*prV2* is found in *D. nodosus* strains capable of causing virulent footrot, while *aprB2* is associated with benign footrot (7, 9). The genetic basis for the difference in disease severity is a 2-base pair change, which results in a single amino acid difference from tyrosine in the secreted AprV2 protease, to arginine in AprB2, located at the tip of the “I2” loop (10, 11). This conveys a difference in thermostability of the proteases, with AprV2 showing increased stability in heat (11).

Real-time polymerase chain reaction (rtPCR) methods have been developed for the detection of *D. nodosus*, and the identification of *aprV2/aprB2*, with results from foot swabs obtained within a day (12, 13). These molecular methods can detect co-infection and quantitate bacterial loads. In comparison, culturing methods are more labor intensive and require specialist media, taking several weeks for a result (3). Current research shows foot scores 1 and 2 present with the highest bacterial load of *D. nodosus* (12, 14, 15). This suggests early detection of *D. nodosus*, in particular, those strains possessing *aprV2*, could have management implications, with molecular methods helping to make informed and timely disease management decisions. Detection of infection early, prior to severe disease symptoms, may help with preventing progression to virulent footrot. Although an rtPCR can provide a quick confirmation of diagnosis, these assays are still not capable of being used in-field for real-time clinical decision making.

An in-field assay for *aprV2*-positive *D. nodosus* has been developed using loop-mediated isothermal amplification (LAMP) (16). LAMP has consistently been shown to be tolerant to biological substances that commonly inhibit conventional or rtPCR assays, such as serum, plasma, urine, aqueous humics, feces, and vitreous (17). In addition to this, LAMP is robust and can tolerate extended periods of warming, 2 pH unit changes, and 10°C changes in operating temperatures (18). With these properties, LAMP is consistently identified as suitable for field use, however, few developed methods have been reported to progress to in-field use (19, 20).

A LAMP for the detection of *aprV2*-positive *D. nodosus* (VDN LAMP) has been developed and a pilot study for in-field use reported previously (16). Using this method, a field trial on 19 sheep properties across Victoria, Australia, has been conducted. Here we report the results of the field trial, comparing VDN LAMP to *aprV2/aprB2* rtPCR, using 309 sheep. We report recommendations for optimized performance when sampling and use in-field.

## Materials and Methods

### Control Strains

Genomic DNA (gDNA) was extracted from cultured cells of *D. nodosus* isolate A198 (*aprV2* positive) (AC: 6466) using PrepMan® Ultra Sample Preparation (Life Technologies) as per manufacturer's instructions. The *D. nodosus* isolate gDNA extraction was used as a positive control throughout and was provided by DAFWA Diagnostics and Laboratory Services (Department of Agriculture and Food Western Australia).

### Field Sample Preparation

A series of field sampling methods were screened in the laboratory before field use (Supplementary Data 1). The final method chosen for sample collection and processing was a dry swab (CLASSIQSwabs, Copan Italia), taken from the interdigital space, or if a lesion was present, the lesion edge and collected into 500 μL alkaline polyethylene glycol (PEG) at pH 13.0.

A sampling matrix was re-created to determine an appropriate dilution for use, using purpose collected sheep samples, confirmed by *aprV2/aprB2* rtPCR (12) as *D. nodosus* negative. Buffer from 5 samples was pooled and aliquoted in 500 μL volumes to distribute inhibitors evenly, before artificial spiking with A198 *D. nodosus* broth (3). A 10 times cell dilution series of 2.05 × 10^8^ cells/mL was made, before spiking 50 μL into the buffer. A secondary dilution series of buffer was made, and 5 μL used directly as rtPCR template for the screening of an appropriate dilution for field use. Cell numbers were calculated using OD600 calculations of neat broth. All cell/buffer dilution combinations were tested using the *aprV2/aprB2* rtPCR (12). Dry swabs collected into 500 μL alkaline PEG, pH 13.0 and a 1:100 dilution of the crude extract was subsequently used as VDN LAMP template throughout the study.

### Sample Collection

This study was carried out in accordance with the recommendations of the La Trobe University animal ethics committee. The protocol was approved by the La Trobe University animal ethics committee with approval number AEC17-21. VDN LAMP field swabs were collected from August to December 2017. Properties were convenience sampled. Current clinical foot scores (Table 1) of the sheep feet were recorded and the single highest scored foot was sampled. Two swabs per sampled sheep were collected simultaneously as above and used as biological duplicates. Per property, 14 sheep were sampled. One swab was used for *aprV2/aprB2* rtPCR and the second for immediate in-field processing with VDN LAMP.

**Table 1:** Modified Egerton foot scoring used to class clinical signs of footrot.

**CLINICAL FOOT SCORE**	
**Score**	**Description**
0	Normal foot with no lesion.
1	A limited mild interdigital dermatitis.
2	A more extensive interdigital dermatitis.
3	Severe interdigital dermatitis and under-running of the horn of the heel and sole.
4	Severe interdigital dermatitis and under-running of the horn of the heel and sole but with the under-running extending to the walls of the hoof.
5	Necrotizing inflammation of the deeper epidermal layer (laminae) of the abaxial wall with under-running of the hard horn of the hoof wall.

Swabs for *aprV2/aprB2* rtPCR were collected into 600 μL phosphate buffered saline (PBS) with 20 mM ethylenediaminetetraacetic acid (EDTA), pH 8.0. Swab heads were snapped into the buffer tubes and were not removed, with transport at 4°C before processing in the laboratory.

Swabs for in-field processing with VDN LAMP were placed into 500 μL alkaline polyethylene glycol, pH 13.0. Swab heads were snapped into the buffer tubes and left in, with collection and processing occurring at ambient temperature.

### In-Field *D. nodosus* Strain Typing Using VDN LAMP

VDN LAMP swabs were left in collection buffer for a minimum of 10 min at ambient temperature, ranging from ~6 to 35°C, before template preparation. The template was prepared by diluting the alkaline PEG containing the swab, pH 13.0, 1:100, using a disposable 10 μL inoculation loop and placing the loop of liquid into 990 μL H_2_O in a microfuge tube. Microfuge tubes were then shaken and 5 μL of this dilution was added via pipette directly to VDN LAMP reaction mixture as a template.

VDN LAMP reactions were carried out as follows; 25 μL total volume using 15 μL OptiGene GspSSD2.0 Isothermal Mastermix (ISO-DR004), 5 μL primer mix (final concentrations of 1.6 μM FIP and BIP, 0.2 μM F3, B3, and LF) (Bioneer), and 5 μL template. Primers used are as previously described (Table 2) (16). Aliquots of all reagents were prepared prior to the property visit in volumes required for one property and were transported in microfuge tubes in a cooler box with ice blocks. Mixing of reagents in-field was performed with a pipette. Transport times ranged from 4 to 10 h.

**Table 2:** Primer sequence and corresponding LAMP primer design region on sequence.

**Primer**	**Primer sequence 5^**′**^-3^**′**^**
FIP	TAACCACCGCATGCCCAGTTATCAAACCAGTCGCAATAGCCA AATTTCTTTAGATGG
BIP	TATCCTGATCCACGCAAAGAAAGAAGCGGTTATTGGTTA CCGCAGC
F3	CGTTTTACCAGGTTATGACTT
B3	CACCAGCAACACCGATAC
LF	TCAGCATCGCGACCATCA

*Underlined regions indicate sequence is complementary to 5′-3′ sequence regions*.

VDN LAMP was run on the Genie II (Optigene, UK) real-time fluorometer, with the following conditions; 40°C preheat for 60 s, 65°C for 20 min, annealing from 94 to 84°C at a rate of 0.5°C/s. Results are reported as the time to positive (Tp) (minutes.seconds) and anneal melting temperature (Tm) (°C), given when the sample fluorescence crossed the pre-set machine threshold of 0.010 fluorescence units.

A run was considered valid if the A198 (*aprV2*-positive) control strain gDNA amplified before 13 min and gave a Tm within the range of 87.7 and 88.7°C, and the no template control (MilliQ H_2_O) showed no amplification. A sample was considered positive if the following criteria were met; a result of both a Tp of ≤20 min and a Tm within 87.7 and 88.7°C.

### Nucleic Acid Extraction and rtPCR for *D. nodosus* Strain Typing

Using 50 μL of the 600 μL buffer, samples for rtPCR had all nucleic acids present extracted and purified using the MagMax Viral RNA extraction kit (Thermo Fisher Scientific) and Kingfisher-96 magnetic particle handling system (Thermo Fisher Scientific) as per manufacturer's instructions. The presence of *aprV2* and/or *aprB2* in samples were identified using primers, probes and cycling conditions as described by Stäuble et al. (12). The AgPath-IDTM One-Step RT-PCR Kit (Ambion, Austin, USA) was used as master mix according to manufacturer's instructions, adapted for 10 μL final volume. Primers and probes were synthesized and supplied by Applied Biosystems (California, USA). Reactions and analysis were carried out on the Mic qPCR Cycler (BioMolecular Systems, Queensland, Australia), using auto threshold detection and bulk analysis.

### Statistics

Statistics were performed using Microsoft Excel 2016 and GraphPad Prism 6. The following criteria were applied to all results;

- rtPCR positive samples have Ct values of under 35 (21)
- rtPCR *arpV2* positive samples have *aprV2* present, either singularly or in a mixed infection with *aprB2*
- rtPCR *aprV2* negative samples have *arpB2* only present, or are *D. nodosus* negative
- VDN LAMP positive samples showed both a Tp before 20 min and a Tm between 87.70 and 88.70°C at the time of processing in-field

The level of agreement between rtPCR and VDN LAMP was evaluated using Cohen's kappa coefficient from Fleiss and Levin (22) and interpreted using the strength of agreements of the Altman scheme where ≤0 = worse than chance alone, <0.20 = poor, 0.21–0.40 = fair, 0.41–0.60 = moderate, 0.61–0.80 = good, and 0.81–0.99 = very good, 1.00 = perfect. The Pearson correlation coefficient was used to measure the linear correlation between Tp and the Ct value of paired samples.

To compare in field VDN LAMP to lab-based rtPCR, VDN LAMP sensitivity (Se) is defined as the percentage of VDN LAMP positive samples within rtPCR *aprV2* positive samples, while VDN LAMP specificity (Sp) is defined as the percentage of VDN LAMP negative samples within rtPCR *aprV2* negative samples. Se, Sp, were calculated using GraphPad Prism 6, along with the negative predictive value (NPV) and positive predictive value (PPV).

Property details including breed, age, and sex of sampled sheep were recorded, in addition to the physical properties of samples, the date and ambient temperature of the day of sampling.

**Figure 1:**
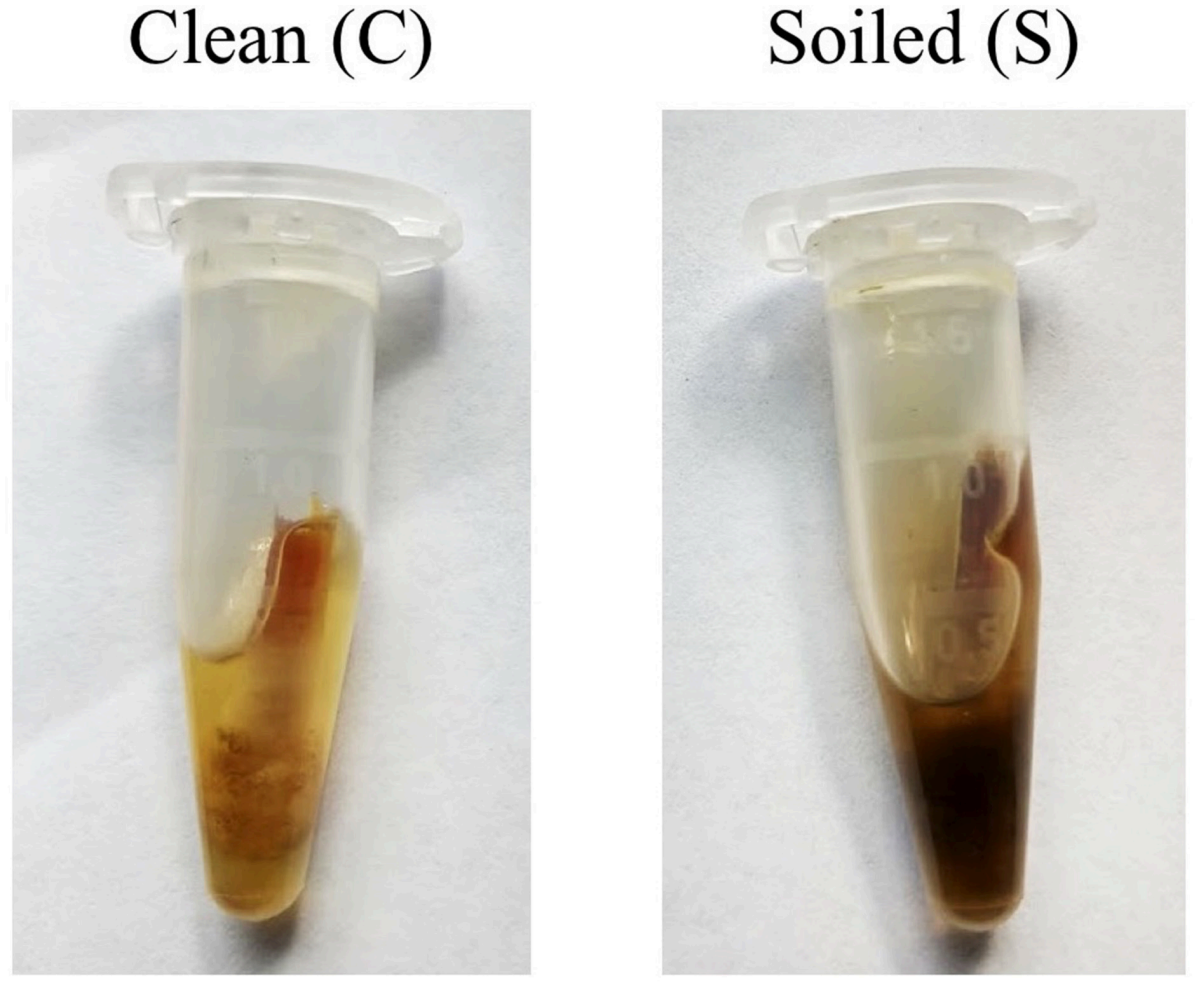
Examples of what was considered a “clean” and “soiled” sample. Care was taken to try and minimize the amount of particulate collected on the swab before placement into buffer.

VDN LAMP results were also analyzed against rtPCR results for sensitivity and specificity with the following parameters;

- Sample moisture (SM) for each property. SM was designated as follows; moist (M), where moisture was present in the interdigital skin, either through the environment or being “sweaty,” as seen when clinical symptoms are present, and dry (D), where the interdigital skin had no moisture present, either from the environment or clinical symptoms.
- Presence of dirt when sampling for each property; clean samples had minimal dirt and feces present (C), while soiled samples (S) had enough dirt and feces to collect onto the swab and significantly color the buffer (Figure 1).
- Property clinical status. Sheep properties were designated as follows; virulent (V), where there are clinical foot scores 4+ present on the property; benign (B), where there are scores of 1 and 2, with this including two sheep properties where 1/14 sheep sampled were scored 3; and negative (N), where all foot scores are 0.
- Ct value ranges of paired swabs, with results grouped as Ct <25, 25 ≤ 30, and 30 ≤ 35.

## Results

### Field Sample Preparation

From the nine methods investigated for field processing of samples, a swab collected into 500 μL alkaline PEG, pH 13.0, was the easiest sampling method to perform with the least amount of equipment. FTA cards produced minimal positive results, while the other swab methods worked well yet proved to be inconvenient or did not show a positive result (Supplementary Data 1). Using 1:100 diluted alkaline PEG, pH 13, directly as the template, a sensitivity of ~1,000 *D. nodosus* cells was able to be detected at a Ct of 35 (Table 3), as determined by rtPCR. A 1:100 dilution of the swab extract provided positive Ct values across a range of cell dilutions and is recommended by the authors of the method to reduce the pH to a more favorable level for DNA amplification (23), while also diluting inhibitors.

**Table 3:** Ct values when using the *aprV2/aprB2* rtPCR and various *aprV2* positive *D. nodosus* cell concentrations diluted into alkaline PEG buffer (pH 13), and subsequent buffer dilutions into H_2_O.

**Cell number**	**Buffer dilution factor**				
	**Neat**	**1:10**	**1:100**	**1:1,000**	**1:10,000**
1,025,000		25.9	24.9	27.6	30.4
1,02,500			29	32	25
10,250			32		38.4
1,025		31	35		
100	37	35	37		

*Crude extract of the dilutions were directly used as rtPCR template. The Ct value of each dilution is given in the corresponding column/row*.

### All Data—*D. nodosus* Strain Typing In-Field, Sensitivity, Specificity, Positive, and Negative Predictive Value

The sensitivity, specificity, positive predictive value (PPV), and negative predictive value (NPV) of the in-field VDN LAMP was calculated by direct comparison with the *aprV2* rtPCR Ct values as determined from the biologically duplicate swabs.

The in-field processing with VDN LAMP gives a sensitivity of 59.62% (Table 4), detecting 93 of the 156 samples that were *aprV2* rtPCR positive. A specificity of 97.39% is seen for all data, with 4/153 samples that were *aprV2* negative testing positive using the VDN LAMP. There is “moderate” agreement between the two methods when comparing *aprV2* positives using Cohen's Kappa coefficient.

**Table 4:** Sensitivity, specificity, and corresponding positive and negative predictive values when comparing identification of *aprV2* presence between the *aprV2/aprB2* rtPCR and VDN LAMP assays on biologically duplicate samples.

	**rtPCR *aprV2* +**	**rtPCR *aprV2*-**	**Total**
VDN LAMP +	93	4	97
VDN LAMP −	63	149	212
Total	156	153	309
Sensitivity	59.62%	95% CI 51.47–67.39%	
Specificity	97.39%	95% CI 93.44–99.28%	
Positive predicative value	95.88%	95% CI 89.76–98.87%	
Negative predicative value	70.28%	95% CI 63.64–76.35%	
Cohen's kappa	0.568	95% CI 0.483–0.653	
Agreement	Moderate		

### Optimizing Sampling Conditions In-Field for VDN LAMP Performance

Some of the likely variables to be encountered during in-field sampling were investigated for effects on assay performance. Sample quality (presence/absence of moisture and dirt), current designation of clinical footrot on the property, and various combinations of these variables were chosen to assist in optimizing sampling procedures in the context of footrot and season of sampling.

#### Sample Quality (Moisture and Presence of Dirt)

The first sampling factor investigated was the presence (moist, M) or absence (dry, D) of moisture in the interdigital space or lesion when collecting the sample. When sampling with moisture present, the sensitivity of VDN LAMP when compared to the *aprV2/aprB2* rtPCR is 83.82%, compared to 39.13% when no moisture is present (Table 5).

**Table 5:** The sensitivity and specificity of the VDN LAMP when compared to the *arpV2/aprB2* rtPCR of moist (M) or dry (D) samples collected from 309 individual sheep.

**SM**	**Sheep properties (n)**	**Sheep (n)**	**rtPCR *aprV2* + (n)**	**VDN LAMP + (n)**	**Se (%)**	**Sp (%)**	**Cohen's kappa (agreement)**
M	7	127	68	59^a^	83.82	96.61	0.796 good
D	12	182	92	38^b^	39.13	97.78	0.367 fair

a 2 samples VDN LAMP false positive.

b *2 samples VDN LAMP false positive*.

The sensitivity of VDN LAMP was reduced in the presence of excessive contaminants in the sample (Table 6). Clean (C) samples have minimal dirt and feces present, while soiled (S) samples had significant contaminants being collected onto the swab. Samples that were both moist and clean (MC) gave an increased sensitivity of 88.89% for the VDN LAMP when compared to the *aprV2/aprB2* rtPCR. Moist and soiled (MS) samples had a sensitivity of 73.91%, while dry and clean (DC) samples a sensitivity of only 39.58% and dry and soiled samples (DS) had a sensitivity of 38.63%.

**Table 6:** The sensitivity and specificity of the VDN LAMP when compared to the *arpV2/aprB2* rtPCR of moist and clean (MC), moist and soiled (MS), dry and clean (DC), or dry and soiled (DS) samples collected from 309 individual sheep.

**Sample quality**	**Sheep properties (n)**	**Sheep (n)**	**rtPCR *aprV2* + (n)**	**VDN LAMP + (n)**	**Se (%)**	**Sp (%)**	**Cohen's kappa (agreement)**
MC	4	86	45	41^a^	88.89	97.56	0.861 very good
MS	3	41	23	18^b^	73.91	94.45	0.664 good
DC	7	112	48	19	39.58	100	0.428 moderate
DS	5	70	44	19^c^	38.63	92.31	0.259 fair

a One sample false positive.

b One sample false positive.

c *Two samples false positive*.

#### Clinical Status of Sheep Properties

VDN LAMP sensitivity increases as the clinical severity of footrot increases, when based on property designation. Those sheep properties considered “negative” (N) for footrot had a sensitivity of 44.44% when compared to the *aprV2/aprB2* rtPCR, while a small difference in sensitivity was observed between a “virulent” (V) designation and a “benign” (B) designation (65.15 and 56.79%, respectively). The “virulent” sheep properties had a lower specificity (88.24%), when compared to the “benign” sheep properties (99.03%) (Table 7).

**Table 7:** The clinical property designation (V, virulent, B, benign, N, negative), number of animals within the property designation and the calculated sensitivity and specificity of VND LAMP in comparison to *aprV2/aprB2* rtPCR.

**Designation**	**Sheep properties (n)**	**Sheep (n)**	**rtPCR *arpV2* + (n)**	**VDN LAMP + (n)**	**Se (%)**	**Sp (%)**	**Cohen's kappa (agreement)**
V	5	83	66	45^a^	65.15	88.24	0.366 fair
B	11	184	81	47^b^	56.79	99.03	0.584 moderate
N	3	42	9	5^c^	44.44	96.96	0.500 moderate

a 2 samples VDN LAMP false positive.

b 1 sample VDN LAMP false positive.

c *1 sample VDN LAMP false positive*.

When both the clinical designation of the property and the moisture status are considered (Table 8), only a slight increase in sensitivity is seen between the benign and virulent groups when sampling “moist” samples (88.24, 83.34%), and similarly poor sensitivity is seen in the “dry” sample groups (42.11, 33.33%). Specificity for the VDN LAMP is highest in the benign group, with both moist and dry sampling, showing 100 and 98.55% specificity, respectively.

**Table 8:** The calculated sensitivity and specificity of VND LAMP in comparison to *aprV2/aprB2* rtPCR when combining clinical property designation (V, virulent, B, benign, N, negative), and the sample moisture, where moisture is present (M), or absent (D).

	**Negative**			**Benign**			**Virulent**		
	**Sheep (n)**	**Se (%)**	**Sp (%)**	**Sheep (n)**	**Se (%)**	**Sp (%)**	**Sheep (n)**	**Se (%)**	**Sp (%)**
M	14^a^	0	92.30	58	88.24	100	55	83.34	92.31
D	28	50	100	126	42.11	98.55	28	33.33	75

a *1 sample false negative, and 1 false positive from the same flock*.

### Specificity of In-Field VDN LAMP and Ct Range Variations in Sensitivity

The four VDN LAMP false positives (Table 9) were from sheep with a clinical score of 0. There was no apparent sample quality association with the 4 samples.

**Table 9:** VND LAMP false positive sample summary data, with no rtPCR cut offs applied, and VDN LAMP time to positive (Tp) and anneal temperature (Tm) displayed.

**Sample**	**Property designation**	**Sample quality**	**Score**	**rtPCR**	**Ct**	**Tp**	**Tm**
1	V	MC	0	*aprV2*	37	19.15	88.36
2	V	DS	0	negative	–	19.00	88.06
3	B	DS	0	*aprB2*	32.3	18.45	88.16
4	N	MS	0	negative	–	19.15	87.96

All VDN LAMP results from Table 9 fall within the recommended range for rtPCR confirmation (16). Sample 1 is the only instance of an *aprV2* positive rtPCR Ct above 35 being identified by the VDN LAMP. This sample is still considered a false positive due to the Ct 35 cut off that is applied to the *aprV2/aprB2* rtPCR. Sample 2 comes from a flock where 10/14 sheep sampled were rtPCR *aprV2* positive, while sample 3 came from a flock where 3/14 sheep sampled were co-infected with *aprV2* and *aprB2* positive strains. An additional 10 sheep from the same property were *aprB2* positive. Sample 4 was from a property where all sheep were scored 0, and 1/14 samples were rtPCR *aprV2* positive. All samples that were *aprV2* rtPCR negative were all from “soiled” samples.

An increase in sensitivity of the VDN LAMP is seen with a decrease in Ct value of the *aprV2/aprB2* rtPCR (Table 10), when comparing the Ct value of the *aprV2* rtPCR positives with the VDN LAMP from the biologically duplicate swab. The sensitivity of samples with Ct's under 25 increases to 86.0%.

**Table 10:** The number of samples identified as virulent by VDN LAMP from biologically duplicate swabs that were positive for *aprV2* via rtPCR, within different Ct ranges (excluding VDN LAMP false positives).

**Ct range**	**rtPCR *aprV2* + (n)**	**VDN LAMP + (n)**	**Se (%)**
< 25	43	37	86.04
25 ≤ 30	87	54	62.07
30 ≤ 35	26	2	07.69

There is a low correlation between Ct value and VDN LAMP Tp, where the Pearson correlation co-efficient is *r* = 0.3161 (*p* = 0.002).

## Discussion

LAMP technology is still in the early stages of full field deployment, with this study contributing to the understanding of factors that impact performance and the machinations of use in-field. The VDN LAMP reagents and machinery performed as anticipated despite difficulties encountered with in-field use in Australian conditions, such as variation in ambient temperature, ranging from 6 to 35°C, and rain. All in-field runs using the *arpV2* gDNA control amplified in 13 min or under, with a Tm in the acceptable range, and the no template control failing to amplify. An adjustment of the previously acceptable Tm range of 88.0–88.9°C to 87.7–88.7°C (16), was deemed necessary after the collection of more field data provided evidence of this range. The chosen in-field sampling method was easy to perform, did not require any machinery and provided consistent results. Though there are few in-field LAMP methods to directly compare to VDN LAMP performance, a study using an in-field laboratory and a real-time LAMP assay for malaria detection reported the sensitivity of 94.1% and specificity of 83.9%. This was using a sample preparation method that included the use of heat treatment, a vortex, centrifuge and aspiration (24), which is significantly more resource intensive than the chosen method presented here. Another study reported a quantitative LAMP for the detection of *Erysiphe necator* (powdery mildew), in-field and performed by producers (25). This study identified a loss of sensitivity over time when used by growers, with samples (*n* = 73) collected using custom impaction spore samplers, and prepared with Chelex 100, boiling, and a vortex. The authors were not able to identify the source of sensitivity loss, with technical aspects of the assay investigated.

When the *arpV2/aprB2* rtPCR and VDN LAMP positive cut-off parameters as defined in the methods are applied, the overall sensitivity of in-field VDN LAMP is 59.62%, correctly identifying 93/156 *aprV2* positive samples, with a specificity of 97.39% (149/153) and a “moderate” agreement to the rtPCR results. The PPV of 95.88% indicates that the presence of a positive sample is reliable, while due to the sensitivity, NPV of 70.28% indicate that a negative result is less reliable. Several factors appeared to influence the sensitivity of in-field testing, with both physical differences in sample and performance between sheep properties noted early on. Of the many factors that can influence a field test, the following variables were investigated for impact on performance; sample moisture, sample cleanliness and the clinical footrot designation of the property. These were chosen as they are important factors when considering technical aspects of LAMP and the context of footrot.

A difference in performance was seen when moisture on the swab is present/absent. Those samples that were “moist” and “clean” showed the highest sensitivity (88.89%) and second highest specificity (97.56%). In contrast, samples that were “dry” and “soiled” had both the lowest sensitivity and specificity (38.63, 92.31%). It should also be noted that those samples that were “dry” and “clean” had similar sensitivity and specificity (39.58, 100%) to “dry” and “soiled.” In addition agreement between the in-field VDN LAMP and the lab based rtPCR was “very good” for moist samples, before lowering to “fair” for “dry” and “soiled” samples. These results suggest that the presence/absence of moisture when sampling is more critical to the success of the assay than the presence/absence of detritus.

It was noted during sampling that interdigital skin or lesions where moisture was present collected visibly more exudate than those where moisture was not present, both making it easier mechanically to perform sampling, but also often indicative of the start of clinical footrot disease symptoms. Recent research has shown that the highest *D. nodosus* load is found with scores 1 and 2, associated with interdigital dermatitis or benign footrot, which are clinically identical (14, 26, 27). The implication for VDN LAMP being that the early stages of infection are likely to produce the best results, through the increased number of bacteria present (increasing sensitivity as more DNA template is present) and the early clinical symptoms producing a moist exudate that contributes to the presence of moisture in the interdigital skin for ease of sampling. The environmental conditions that provide moisture are also those that are more favorable for the growth of *D. nodosus*, which requires the presence of moisture to cause disease (28). In addition, moisture presence/absence is influenced by the weather, suggesting the time of year when sampling will have an impact on the performance of the assay, with sampling over dry months not recommended.

On sheep properties designated virulent, the VDN LAMP had a sensitivity of 65.15% with this reduced to 56.79% for benign sheep properties, and further still to 44.44% on negative sheep properties. When considering disease status of the property, VDN LAMP agreement ranged from “fair” to “moderate,” as assessed by the Altmann scale interpreting Cohen's Kappa coefficient. If the presence/absence of moisture is again considered alongside clinical designation, increases to sensitivity are seen for both benign (88%) and virulent (83.34%) sheep properties. This supports the importance of the presence of moisture when sampling, and the benefit of sampling with VDN LAMP in the earlier stages of disease. The improved performance for, at that current time of sampling, “benign” sheep properties, could be beneficial if VDN LAMP is to be used as a tool for the identification of risk for developing severe footrot via detecting *aprV2* positive *D. nodosus*. This type of risk identification approach may be beneficial for sheep producers and veterinarians who wish to prevent any further disease development, based on VDN LAMP results, climatic conditions and individual flock characteristics. It has been suggested that using *D. nodosus* characteristics rather than clinical expression could improve control outcomes (29), with VDN LAMP and *aprV2* detection a potential tool for this type of approach. The elimination of *aprV2* positive *D. nodosus* has been previously demonstrated as possible in Swiss sheep flocks (30).

Of the 309 samples tested, 4/153 samples were in-field VND LAMP positive and rtPCR *aprV2* negative. These samples have come from four different sheep properties, with individual sample details listed in Table 9. Of these, sample 1 was *aprV2* rtPCR positive, with a Ct value of 37, which is above the Ct cut off for positive samples. This sample came from a currently virulent footrot property and is the only example to date of VDN LAMP detecting a sample with a Ct above 35. Of the other samples, 1/4 was positive for *aprB2* (sample 3), and comes from a property designated benign. This property had 3/14 samples *aprV2* and *aprB2* positive by rtPCR (co-infection), with 13 samples positive for *aprB2*. The remaining 2/4 VDN false positive samples had no *D. nodosus* detected by rtPCR and come from a clinically negative (sample 4) and a clinically virulent sheep property (sample 2). The sheep property designated virulent had 10/14 samples rtPCR *aprV2* positive, with 3/10 correctly identified by VDN LAMP. The sheep sampled in this instance had typically overgrown hooves, with large amounts of dirt present in the overgrowth, which increased sampling difficulty. It has also been reported previously that in severe lesions that are deep under the horn, *D. nodosus* is more difficult to access as it resides deep in the lesion. It is possible there was not enough bacteria, and too much soil, for adequate performance of the VDN LAMP, whereas being more stringent and sensitive, the rtPCR was able to identify the *aprV2* positive samples. There was 1 sample from a negative property that was rtPCR *aprV2* positive and VDN LAMP negative, and a false positive detected within another sample. There is the potential that the 4/153 samples were cross contaminated, but as all samples had Tp's above 18 min, they are within the recommended range for additional testing to confirm results (16). It is also possible that variation between the two biological duplicate swabs exists and has contributed to the variation seen, however there is no way to investigate this retrospectively. Swabs were collected simultaneously with all efforts made to minimize the variation.

As reported previously (16), VDN LAMP's sensitivity in the laboratory increases with a decrease in Ct values of the *aprV2/aprB2* rtPCR, and this is true also in-field with paired samples. There is however only a low correlation between the Ct value and Tp, indicating that quantitative analysis from VDN LAMP would not be accurate. The ability to quantitate from LAMP is a source of discussion in the literature (31), and with the results presented here, the authors believe when used in-field the presence/absence of the target is an appropriate interpretation of results. To contrast detection limits for *D. nodosus* between common methods, it was recently reported that culturing has a detection limit of ~1,000 *D. nodosus* cells, whereas rtPCR is around 10 cells (32). VDN LAMP falls between this range, detecting approximately 950 *D. nodosus* cells when estimated using a Ct 30 from biologically duplicate swabs, or 40 cells when considering the lower concentration end of detection, at Ct 35.

That there is no correlation between Tp and bacterial load, plus the variation in VDN LAMP sensitivity seen between sheep properties, the authors suggest the interpretation of results should take into account the proportion of positives in the group and the time of sampling—a single positive on a property with moisture present warrants further investigation using more sensitive methods, or a larger sample size, with VDN LAMP providing a basic screening of the flock. However, if a large portion of the samples are VDN LAMP positive, regardless of Tp, *aprV2* does appear to be present in the flock. The 3/4 samples that did not show an rtPCR *aprV2* signal, yet were VDN LAMP positive, all came from flocks where there was evidence of *arpV2* and varying clinical symptoms of footrot. A flock level interpretation of results is appropriate for typical management practice in Victoria, where traditionally whole flocks are footbathed as a group for treatment.

## Conclusion

The VDN LAMP is a new addition to the suite of diagnostics for footrot and is capable of use in-field. This assay offers the fastest time to results, within 1.5 h, which is significantly faster than the well-established culturing methods, and the newer molecular tests. The time taken for an rtPCR result, once samples are transported via cool chain to a laboratory, is 1 day, while culturing routinely takes up to 4 weeks for a virulence result. The VDN LAMP performs best when sampling occurs with moisture present and minimal dirt, with the presence of moisture corresponding to the environment that is ideal for *D. nodosus* proliferation and expression of virulence factors in Victoria. Sampling at the end of winter/start of spring, which is the traditional time for footrot spread in Victoria, is recommended for increased sensitivity. If used as recommended, the sensitivity of the VDN LAMP is 89% and specificity 97%. Test results in the above context should consider the number of VDN LAMP positives identified and the clinical signs of footrot on the property. Rapid identification of *aprV2* positive *D. nodosus* infection in-field may help reduce spread of footrot through earlier detection, encourage more preventative or new management strategies, and provide evidence or confirmation of infection. Advantages of VDN LAMP in-field for *aprV2* positive *D. nodosus* detection in flocks includes having information of infection in real time, and therefore informing decisions about stock movements and treatment.

## Author Contributions

NB performed the experiments and wrote the manuscript. GR, RS, BR, and TB contributed reagents, materials, and/or analytical tools and wrote the manuscript.

### Conflict of Interest Statement

The authors declare that the research was conducted in the absence of any commercial or financial relationships that could be construed as a potential conflict of interest.
