# Supplementary material for: Optimization of a Loop Mediated Isothermal Amplification (LAMP) Assay for In-Field Detection of Dichelobacter nodosus With aprV2 (VDN LAMP) in Victorian Sheep Flocks
Source: Front Vet Sci. 2019 Mar 8;6:67. doi: 10.3389/fvets.2019.00067 (PMC6418044; doi:10.3389/fvets.2019.00067)
Supplement: Supplementary file 1 [file Table_1.DOCX]

Supplementary Material

Optimisation of a Loop Mediated Isothermal Amplification (LAMP) Assay for In-Field Detection of *Dichelobacter nodosus* with *aprV2* (VDN LAMP) in Victorian Sheep Flocks

**Nickala Best^1^, Grant Rawlin^2^, Robert Suter^3^, Brendan Rodoni^2^, Travis Beddoe^1*^**

*** Correspondence:** Dr. Travis Beddoe, t.beddoe@latrobe.edu.au

# Supplementary methods and data

Swabs (CLASSIQSwabs) or Whatman FTA cards (Sigma Aldrich) collected from 5 individual sheep were placed into 500 µL of various buffers (Table 1) to re-create the sampling matrix, before spiking with *Xanthamonas vesicatoria* (BRIP 62429). Spike samples were screened for *X. vesicatoria* using rtPCR, adapted from conventional PCR as described in Koenraadt et al. (2009) to determine if the sample preparation method had worked. Swabs were collected as previously described (Best et al., 2018). Whatman FTA cards had two folds made, creating a 1.5 cm central channel that was swiped through the interdigital skin, collecting material onto the channel face before taking a punch of the card containing material. Each buffer type with sample was pooled, and re-aliquot to evenly distribute inhibitors. A series of *X. vesicatoria* cell dilutions (10^6^ – 10^2^ cells) were spiked into each buffer type, before a second series of dilutions of the buffer (neat, 1:10, 1:100, 1:1,000, 1:10,000) was made into H_2_O to identify the most suitable dilution for inhibitor removal. An additional FTA punch for sampling methods 6 – 8 had 15 µL of 5x10^8^ *X. vesicatoria* cell suspension added before being placed into buffer, and were only placed in the neat sample before dilution.

Table 1; Methods of sample collection, including collection substrate, buffer, and treatment to sample if applicable.

| Sampling method | Collection | Buffer | Treatment |
| --- | --- | --- | --- |
| 1 | Swab | Phosphate buffered saline (PBS) with 20 mM EDTA, pH 8 | Boiled |
| 2 | Swab | Water | Boiled |
| 3 | Swab | MacKenzie buffer (MacKenzie et al., 1997) | None |
| 4 | Swab | CTAB (100mM Tris-HCL pH 8, 100mM Ethylenediaminetetraacetic acid (EDTA), 100mM NaH_2_PO_4_ pH 8, 1.5M NaCl, 2% (w/v) hexadecyltrimethylammonium bromide (CTAB) and 1% (w/v) Polyvinylpyrrolidone average mol wt 360,000 (PVP360)) | None |
| 5 | Swab | CTAB + 100 µM Aluminium | None |
| 6 | Swab | Alkaline PEG pH 13 (Chomczynski and Rymaszewski, 2004) | None |
| 7 | FTA punches | Alkaline PEG pH 13 (Chomczynski and Rymaszewski, 2004) | None |
| 8 | FTA punches | MacKenzie buffer (MacKenzie et al., 1997) | None |
| 9 | FTA punches | 0.5X TBE | None |

# Results

Table 2: Cell and buffer dilutions of sampling methods 1 – 9 used directly as rtPCR template and the corresponding Ct value from *X. vesicatoria* specific rtPCR.

|  | **Buffer dilution factor** | | | | |
| --- | --- | --- | --- | --- | --- |
| Sampling method 1 (swab in PBS with 20 mM EDTA, boiled) | | | | | |
| **Cell number** | **Neat** | **1:10** | **1:100** | **1:1000** | **1:10000** |
| 1000000 |  | 25.1 |  | 18 | 17.8 |
| 100000 |  |  | 25.4 | 28.4 | 24.4 |
| 10000 |  |  | 29 |  |  |
| 1000 |  |  | 36 |  |  |
| 100 |  |  |  |  |  |
| Sampling method 2 (swab in H_2_O, boiled) | | | | | |
| 1000000 |  |  | 4 | 21 | 22 |
| 100000 |  |  | 25 | 31 |  |
| 10000 |  |  | 31 |  |  |
| 1000 |  |  |  |  |  |
| 100 |  |  |  |  |  |
| Sampling method 3 (swab in MacKenzie buffer) | | | | | |
| 1000000 |  |  |  | 33.7 | 36.9 |
| 100000 |  |  | 37 |  |  |
| 10000 |  |  |  |  |  |
| 1000 |  |  |  |  |  |
| 100 |  |  |  |  |  |
| Sampling method 4 (swab in CTAB) | | | | | |
| 1000000 | 29.57 |  |  |  |  |
| 100000 |  |  |  |  |  |
| 10000 |  |  |  |  |  |
| 1000 |  |  |  |  |  |
| 100 |  |  |  |  |  |
| Sampling method 6 (swab in alkaline PEG, pH 13) | | | | | |
| 1000000 |  |  | 30.8 | 33.6 |  |
| 100000 |  |  | 36.67 |  |  |
| 10000 |  |  |  |  |  |
| 1000 |  |  |  |  |  |
| 100 |  |  |  |  |  |
| Sampling method 7 (FTA punch in alkaline PEG, pH 13) | | | | | |
| 3000000 |  | 36 | 38 |  |  |
| 300000 |  |  |  |  |  |
| 30000 |  |  |  |  |  |
| 3000 |  |  |  |  |  |
| 300 |  |  |  |  |  |
| Sampling method 9 (FTA punch in 0.5X TBE) | | | | | |
| 3000000 |  | 36 | 37 |  |  |
| 300000 |  |  |  |  |  |
| 30000 |  |  |  |  |  |
| 3000 |  |  |  |  |  |
| 300 |  |  |  |  |  |

Swabs collected into CTAB with Aluminium (Sampling method 5) and FTA punches collected into MacKenzie buffer (Sampling method 8) showed no positives.

# References

Best, N., Rodoni, B., Rawlin, G., and Beddoe, T. (2018). The development and deployment of a field-based loop mediated isothermal amplification assay for virulent Dichelobacter nodosus detection on Australian sheep. *PLOS ONE* 13**,** e0204310.

Chomczynski, P., and Rymaszewski, M. (2004). Alkaline polyethylene glycol-based method for direct PCR from bacteria, eukaryotic tissue samples, and whole blood. *BioTechniques* 40**,** 454-458.

Koenraadt, H., Van Betteray, B., Germain, R., Hiddink, G., Jones, J.B., and Oosterhof, J. (Year). "Development of specific primers for the molecular detection of bacterial spot of pepper and tomato.": International Society for Horticultural Science (ISHS), Leuven, Belgium), 99-102.

Mackenzie, D.J., Mclean, M.A., Mukerji, S., and Green, M. (1997). Improved RNA Extraction from Woody Plants for the Detection of Viral Pathogens by Reverse Transcription-Polymerase Chain Reaction. *Plant Disease* 81**,** 222-226.

**
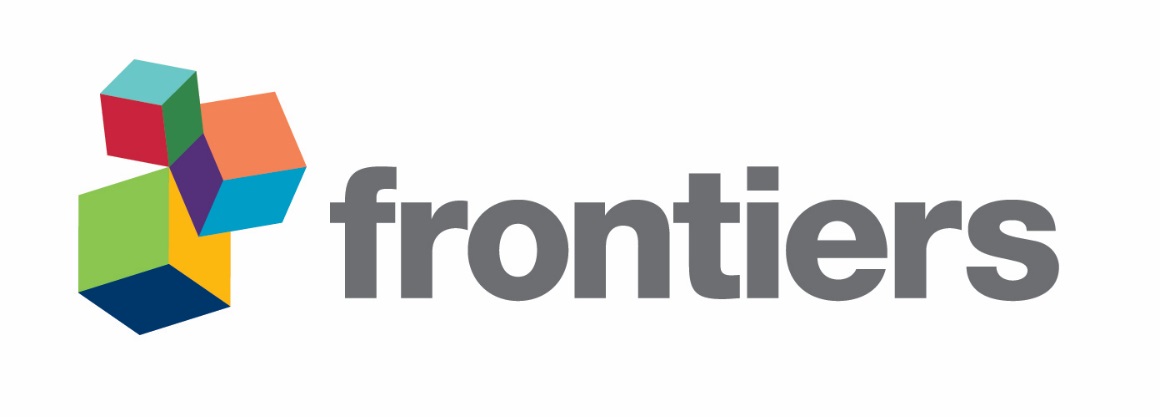
**
